# Supplementary material for: Brief mindfulness coaching enhances selective attention in medical scientists: A pilot study
Source: PLoS One. 2025 Sep 12;20(9):e0330290. doi: 10.1371/journal.pone.0330290 (PMC12431218; doi:10.1371/journal.pone.0330290)
Supplement: S2 File — (DOC) [file pone.0330290.s002.doc]

| UCSD Human Research Protections Program **New Social and Behavioral Sciences Application**  **RESEARCH PLAN**  Version Date 10/01/09  (Enter text in the **white space areas** below each numbered heading bar. **Expand the size of table cells as needed** – to multiple pages if needed.  (See SBS Application Instructions for explanation of headings and information to be provided) |
| --- |
| **1. PROJECT TITLE** |
| T32 Wellness Supplement |
| **2. PRINCIPAL INVESTIGATOR, FACULTY ADVISOR, SUPERVISOR** |
| Principal Investigator: David M. Roth, MD, PhD |
| **3. FACILITIES** |
| UC San Diego  UC San Diego Health  UC San Diego Health Sciences |
| **4. ESTIMATED DURATION OF THE STUDY** |
| 1 yr |
| **5.** **SPECIFIC AIMS (2 paragraphs maximum)** |
| The T32 supplement is designed to address a need to enhance wellness and resiliency in early physician-scientist trainees/faculty. We will supply wellness/resilience training to trainees in the MSTP program, Anesthesiology T32, and early faculty. We will expand wellness efforts already initiated for MSTP trainees. We will introduce new elements of wearable technology, app based monitoring to stress, and brain electrical activity measurements to add complementary scalable approaches to identify and manage risk in loss of wellbeing. Our work will synergize wellness and resiliency efforts by applying them to a new, important cohort of trainees currently not being directly addressed at UCSD. We propose to undertake a unique, evidence-based effort to understand wellness and resilience using multiple innovative approaches over a 12-month period. We will focus our efforts on 4 groups that experience unique transitions in the education of MSTPs and the development of a research career that are particular triggers of stress and anxiety:  **Group 1)** MSTP students in the first two years of medical school;  **Group 2)** MSTP students transitioning into the PhD portion of their training;  **Group 3)** MSTP students transitioning back to their clinical years 3 and 4 of medical school;  **Group 4)** trainees or recent graduates of the Anesthesiology T32 program and MSTP graduates who are UCSD junior faculty members. |
| **6. BACKGROUND AND SIGNIFICANCE (2-3 paragraphs maximum)** |
| Physician-scientists hold a unique place in academia and society. At academic institutions, they are considered a “triple threat” whereby they provide excellent clinical care, perform cutting edge research, and are impactful educators. In the Hippocratic Oath, physicians are taught ‘to first do no harm’. The outward expression of this oath is to provide compassionate, empathetic care to patients. Unfortunately, the cost of achieving this high standard is the need to constantly balance patient care, research, teaching duties, and home life, each at the expense of the other, potentially leading to the crushing of the individual spirit through inward harm. This dilemma then impacts patient care and physician wellbeing and resilience.  Physician burnout is described as physical and mental exhaustion and is prevalent, involving more than 50% of physicians and trainees.^1-3^ It occurs in both primary and procedure-based physicians throughout the world and at various career stages.^4, 5^ Three core aspects of burnout comprise: emotional exhaustion, depersonalization/cynicism, and feelings of inadequacy and poor accomplishment.^3, 6^ Burnout leads to significant costs in terms of limiting empathetic patient care,^7^ financial impact into the billions of dollars, and is associated with personally destructive behavior, loss of clinical care hours, and turnover of physicians.^8^ Growing evidence suggests that the underpinnings of a lifestyle prone to dysfunctional wellness and resilience may start as early as medical school, given the competitive nature of getting into and succeeding in medical school by learning an immense amount of information (i.e., described by many as drinking from a firehose), performing on exams, matching to optimal residencies, and ultimately assuming the responsibility of patient care.^9^ As such, our T32 supplement involves two NIGMS-funded T32 programs at the University of California, San Diego (UCSD). The first program in the Department of Anesthesiology (*T32GM121318*) is designed to create and train physician-scientists. The second program is a cohort of students in the Medical Scientist Training Program (MSTP, *T32GM007198*) and includes junior faculty that are graduates of the UCSD-MSTP program. We will explore wellness and resilience in individuals from both T32 training programs |
| **7. PROGRESS REPORT/PRELIMINARY STUDIES** |
| N/A |
| **8. RESEARCH DESIGN AND METHODS (1 page maximum)** |
| There are currently 85 UCSD-MSTP trainees, 4 trainees that have completed or are currently in the Anesthesiology T32 program, and more than 10 MSTP graduates that are UCSD junior faculty members. We will recruit 20 trainees for Group 1-3 and 10 junior faculty members for Group 4.  Study procedures for all individuals regardless of groupings:   1. All participants will be provided a Garmin (Vivo Smart 4) as a wearable device to passively gather information (i.e., activity, heart rate, daily resting heart rate, oxygen saturation, sleep, stress). The participants will be encouraged to wear the Garmin as often as possible for the 12 month study duration. The participants will get to keep the Garmin at the conclusion of the study. 2. The study is designed as a crossover where all participants will be involved with meetings with Drs. Beck and Rana to learn about health, wellbeing and resilience, leadership training, and group-based wellness approaches. The sessions will be scheduled every other month for six months (3 sessions total). At the six- month time the second group of participants will be meeting with Drs. Beck and Rana for 3 sessions over the last six months of the study. 2) access to a wellness coach, Dr. Ramanathan, (who is currently involved on an as-needed basis in this capacity in the MSTP) at least once quarterly (more often if needed), 3) interactions with senior faculty mentors (Drs. Patel, Roth, Insel, Chi, and Beck) to develop short, medium, and long-term goals for work and home life, 4) integration of the RAEHealth App coupled to Garmin measures to identify and mitigate stress, and 5) the program faculty, with input from the participants, will identify structural programmatic, institutional, and climate changes that could support wellbeing and advocate for institutional modifications based. All individuals will have access to mindfulness training from the UCSD Center for Mindfulness which is open to all UCSD staff and students.   All individuals will undergo pre and endpoint (12 month) collection of quantitative data, which will be collected by the following measures: 1) Maslach Burnout Inventory 2) Neff Self-Compassion and Compassion Scales 3) Mindful Attention Awareness Scale and 4) 7-item Shortened Warwick-Edinburgh mental well-being scale (SWEMWBS; Tennant R, Hiller L, Fishwick R, Platt S, Joseph S, Weich S, et al. The Warwick-Edinburgh Mental Well-being Scale (WEMWBS): development and UK validation. Health Qual Life Outcomes. 2007;5(63):1–13. These questionnaires will be completed by interview, online, or e-mail. After 12 months, we will conduct a survey of the participants using the following open-ended questions, such as: 1) Rate overall experience on a scale, 1-5. 1 being poor and 5 being excellent. Are there any things you particularly valued, liked or appreciated, or anything you feel could be improved or modified? 2) Overall, what do you see as your greatest learnings? (Please share up to three learnings/useful takeaways from these sessions.) 3) As a result of this experience, do you expect your behavior to change in the future? 4) If so, in what ways? 5) General Comments. The survey will take about one half hour and can be done independently and returned to Dawna Chuss at dchuss@ucsd.edu.   1. At the pre and endpoint times (total of 2 timepoints) participants will be asked to come to the ACTRI for assessments. The project will monitor changes in brain activity to emotional stimuli using the UCSD © Brain Engagement (BrainE) platform developed by UCSD co-investigator Dr. Jyoti Mishra. The platform integrates low-cost, high-resolution semidry electrode electroencephalography (EEG) synced with cognitive activity in both non-emotional and emotional contexts.^10^ Neural data are artifact corrected and processed in physiologically relevant spectral-temporal domains and source localized to unique brain regions using updated neural signal processing methods that are especially useful for noisy real-world settings.^11^ Individuals will partake in the EEG recordings in the context of an emotional bias task,^12^ which Mishra and colleagues have shown to elicit differential response with bias towards negative and threatening stimuli during states of social withdrawal, and bias towards positive emotion stimuli during pro-social states. *These data will provide objective biomarkers complementing the changes in observed wellbeing of the study participants.*   **Assessments of Neuro-Cognition.**  All *BrainE* modules include simple written instructions on the screen and a short practice period prior to the main assessment. The assessments are run in several blocks to minimize participant fatigue. All assessments can be completed within a 1 hour session. Simultaneous to all cognitive assessments, noninvasive neurophysiological signals are recorded from a mobile, wireless electroencephalography (EEG) device. Non-invasive EEG is considered a non-significant risk device. The EEG sensors are placed on the participant’s scalp as a cap that fits non-invasively and snugly on the head, akin to a swim cap. The EEG cap has 21-32 sensors on the head that are either dry or have saline dipped sponges to make contact with the scalp. There is no associated skin irritation. We have extensively tested this mobile EEG set up for ease of use and comfort in participants. There is no separate cloud-based data storage for the device. All device data will be securely uploaded onto our lab cloud-based data server. The results of the EEG will not be used for diagnostic purposes and the results may be shared with the participants upon request.  Overall, we will integrate these various measurements to define scalable techniques to identify those at highest risk for poor wellness and resilience allowing for more in-depth and early intervention at the same time that we strengthen prevention. |
| **9. HUMAN SUBJECTS (2 paragraphs maximum)** |
| The group will consist of trainees and junior faculty from the UCSD Health System. All participants are adults (over the age of 18), consisting of both men and women from diverse ethnic backgrounds. We will recruit 20 trainees for Group 1-3 and 10 junior faculty members for Group 4. (Total 70 participants). |
| **10. RECRUITMENT** |
| Recruitment will be from current trainees involved in the MSTP and Anesthesiology T32 training programs at UCSD. The junior faculty involved in the study will be recruited from individuals that have completed T32 training at UCSD in one of UCSD’s T32 training programs. Subjects will be recruited by the staff in the UCSD MSTP program (Colin King) and Department of Anesthesiology T32 Program (Dawna Chuss). Recruitment will be done by e-mail and also through annual retreat and new MSTP orientation announcements. |
| **11. COMPENSATION FOR PARTICIPATION** |
| Participants will receive a $150 Amazon gift card at the end of the study. Participants will also be allowed to keep the Garmin device after completion of the study. |
| **12. INFORMED CONSENT** |
| Trainees and junior faculty will be asked to sign an informed consent form prior to or participating in the study. |
| **13. ALTERNATIVES TO PARTICIPATION** |
| Alternative to participation is not participating. Participants will still be able to be involved in T32 training, if they choose not to participate in the study. |
| **14. POTENTIAL RISKS** |
| The risks of the study are considered minimal and include the following:   1. Breach of confidentiality. Information will be kept in an encrypted, password protected, HIPPA compliant, cloud based server. Only study investigators will have access to this data. 2. Answering questions that might make them feel uncomfortable (e.g., expressing dissenting views about the program) for fear of retaliation or social judgement . 3. The procedures for EEG recording are considered safe and have been standardized for use in research for decades. The procedures are completely noninvasive, and involve no physical or psychological harm. The tight fit of the EEG cap may cause temporary marks on the skin. Physical discomfort from wearing the device will be minimized by ensuring that it fits well. 4. Boredom and/or fatigue 5. Participation may have risks of triggering suicidal ideation. In this case you will have access to the suicide prevention hotline (800-273-TALK) and crisis intervention at UCSD (24 hr 858 534-3755) and no cost counseling at UCSD through CAPS services (858 534-3755) and/or consultation with Dhakshin Ramanathan, MD. Ph.D., a state-licensed psychiatrist. 6. If a Garmin device is lost we will replace the device if resources allow. There will be no negative consequence to the study participant. |
| **15. RISK MANAGEMENT** |
| To ensure confidentiality, all collected information from the participants will be confidential. Upon enrollment, participants will receive an ID number and assessment data will be linked to this number; only one person will have access to the link between the ID and the name of the wellness director. All paper records (i.e., informed consent forms) will be kept in a locked filing cabinet in a locked research office within a secure building that is monitored 24 hours/day and will only be available to the research team. Consent forms will be maintained separately from hard copies of confidential study assessment data.  We will reassure participants that they can safely provide candid feedback without the fear of retaliation or social judgment if they express dissenting views about the study and encourage them to voice their concerns about the study so that we can attempt to address them in the future program design.  If a participant should become uncomfortable with any procedures, he/she can withdraw from the study at any time. You will be provided access to the services of a faculty member outside the study group (Byron Fergerson MD the Director Wellness in the Department of Anesthesiology at UCSD, 801-824-3538) to anonymously report or discuss any issues or concerns with non-participation, reporting discomfort, embarrassment or concerns for retaliation. If psychological distress or another problem should occur, the PI or a designated backup licensed professional (Dhakshin Ramanathan, MD. Ph.D., state-licensed psychiatrist) will be contacted immediately. Additionally, we will take steps including (1) we provide to all participants an updated, ZIP-code customized list of local mental health programs, and, (2) any disclosure of thoughts, intent, or plans to commit self-harm or suicide are immediately reported to the PI or her designee. If any emergency arises, 911 will be phoned immediately. |
| **16. POTENTIAL BENEFITS** |
| There may not be any direct benefits to you from this process. The investigators will learn more about risk factors and the effectiveness of strategies designed to reduce stress and improve wellness among trainees and junior faculty . The data will be used for research and for program improvement purposes. The results of this study have the possibility of increasing wellness and resilience in healthcare providers to benefit future patients and society. |
| **17. RISK/BENEFIT ASSESSMENT** |
| We do not forecast any risk to participating in the study. On the other hand, benefits of participating in the training are anticipated since the trainees and junior faculty are in a group of individuals at risk of burnout and stress related conditions. |
| **18. QUALIFICATIONS, TRAINING, CULTURAL LITERACY AND ROLES OF THE PI AND RESEARCH TEAM** |
| **David M. Roth, M.D., Ph.D., Principal Investigator**. Dr. Roth is a Staff Physician at VA Healthcare San Diego and a Professor of Anesthesiology at the University of California, San Diego. Dr. Roth serves as the co-director of the Anesthesiology T32 program. Dr. Roth has extensive experience with issues of student wellness and resiliency through service as the Chair of the Student Affairs Committee at UCSD School of Medicine (SOM) and service as a member of the Committee on Educational Policy at UCSD SOM. Dr. Roth has mentored a number of students, fellows, residents, and junior faculty over the years. Dr. Roth will be responsible for the overall coordination and supervision of all aspects of the study. He will serve as a mentor for the supplement and be involved in data analysis and publications.  **Hemal Patel Ph.D., Co-Investigator.** Dr. Patel is a Co-Director for the Anesthesiology T32. He has expertise in data integration and analysis of large data sets. He will serve as a mentor for the supplement and be involved in data analysis and publications.  **Ellen Beck, MD, Co-Investigator.** Dr. Beck will oversee the program and fidelity to make sure the training is in line with the primary goals that it was designed for. She has a successful history of empowering and helping to create supportive environments where people and communities take charge of their lives and achieve wellbeing. Dr. Beck will contribute to data analyses and publications.  **Dhakshin Ramanathan, M.D., Ph.D., Co-Investigator.** Dr. Ramanathan is an Assistant Professor in the Psychiatry Department at UCSD and current wellness champion for the MSTP program. He has been a primary mentor to a number of students. His research expertise is in exploring the neural mechanisms of brain plasticity, and neurophysiological processes associated with behavioral deficits after injury. His work is focused on understanding molecular processes that regulate motor map plasticity during development and normal learning. He was recruited to serve as the Wellness coordinator for the UCSD-MSTP program. He will serve as a Wellness coordinator that will have routine meetings with individuals to discuss and implement wellness and resilience practices.  **Jyoti Mishra, Ph.D., Co-Investigator.** Dr. Mishra is an Assistant Professor in the department of Psychiatry at UCSD. Her research focuses on human cognitive, computational and translational neurosciences, as well as expertise in methods including neuroimaging (EEG and fMRI) and digital health technology implementation. She is the founder of the Neural Engineering and Translation Labs (neatlabs.ucsd.edu) and directs its human research. Her research program focuses on engineering neuroscience evidence-based mental health technologies that have the potential to serve as novel digital therapeutics. Additionally, her laboratory develops methodologies to determine neural mechanisms of cognitive functions in healthy as well as cognitively-impaired populations across the lifespan. She will conduct digital wellbeing studies in individuals.  **Deborah Rana, M.D., Co-Investigator.** Dr. Rana has a broad background in General Academic Pediatrics complete with a local and national faculty fellowship in Medical Education and Educational Scholarship with specific training and expertise in curriculum development and wellness practices such as mindfulness meditation and self-compassion. Her scholarship includes a national on-line curriculum in wellness and resilience developed as a member of a workgroup of the American Academy of Pediatrics. She also as expertise in behavioral survey implementation and analysis. She will develop in conjunction with Dr. Beck a wellness and resilience quarterly workshop to develop the cohorts in the active group.  **Paul Insel, M.D., Co-Investigator.** Dr. Insel is a Distinguished Professor in the Departments of Pharmacology and Medicine at UCSD. He has been the director of the MSTP program sine 1989 (for 32 years) and has seen the program transform over the years. He has an interest in seeing MSTP succeed at every stage of their careers and is uniquely involved in learning about and guiding each individual in the program. He has an interested in developing programs to enhance wellbeing and resilience in this cohort and see them succeed as academic faculty. Dr. Insel will consult on the proposed studies in the T32 supplement and serve as a mentor in the program.  **Neil C. Chi, M.D., Ph.D., Co-Investigator.** Dr. Chi is a Professor in the Department of Medicine at UCSD. He recently started as the co-Director of the UCSD-MSTP program and will take over in the next few years when Dr. Insel retires. They work closely together on student wellness issues and serve as mentors for MSTP student across the training experience. Dr. Chi will consult on the proposed studies in the T32 supplement and serve as a mentor in the program. |
| **19. FUNDING FOR THIS PROJECT** |
| We are applying for Supplemental T32 funding from the NIH. |
| **20. CONFLICT OF INTEREST** |
| N/A |
| **21. BIBLIOGRAPHY (1 page maximum)**  **References**  1. Rotenstein LS, Torre M, Ramos MA, Rosales RC, Guille C, Sen S and Mata DA. Prevalence of Burnout Among Physicians: A Systematic Review. *JAMA*. 2018;320:1131-1150. PMC6233645  2. West CP, Dyrbye LN and Shanafelt TD. Physician burnout: contributors, consequences and solutions. *J Intern Med*. 2018;283:516-529.  3. Nene Y and Tadi P. Resident Burnout *StatPearls* Treasure Island (FL); 2021.  4. Lee RT, Seo B, Hladkyj S, Lovell BL and Schwartzmann L. Correlates of physician burnout across regions and specialties: a meta-analysis. *Hum Resour Health*. 2013;11:48. PMC3849515  5. Dyrbye LN, Varkey P, Boone SL, Satele DV, Sloan JA and Shanafelt TD. Physician satisfaction and burnout at different career stages. *Mayo Clin Proc*. 2013;88:1358-67.  6. Maslach C and Leiter MP. Understanding the burnout experience: recent research and its implications for psychiatry. *World Psychiatry*. 2016;15:103-11. PMC4911781  7. Reynolds M, McCombie A, Jeffery M, Mulder R and Frizelle F. Impact of burnout on empathy. *N Z Med J*. 2021;134:12-20.  8. Han S, Shanafelt TD, Sinsky CA, Awad KM, Dyrbye LN, Fiscus LC, Trockel M and Goh J. Estimating the Attributable Cost of Physician Burnout in the United States. *Ann Intern Med*. 2019;170:784-790.  9. Rajapuram N, Langness S, Marshall MR and Sammann A. Medical students in distress: The impact of gender, race, debt, and disability. *PLoS One*. 2020;15:e0243250. PMC7714351  10. Balasubramani PP, Ojeda A, Grennan G, Maric V, Le H, Alim F, Zafar-Khan M, Diaz-Delgado J, Silveira S, Ramanathan D and Mishra J. Mapping cognitive brain functions at scale. *Neuroimage*. 2020;231:117641.  11. Ojeda A, Klug M, Kreutz-Delgado K, Gramann K and Mishra J. A Bayesian framework for unifying data cleaning, source separation and imaging of electroencephalographic signals. *bioRxiv*. 2019:559450.  12. Grennan G, Balasubramani PP, Alim F, Zafar-Khan M, Lee EE, Jeste DV and Mishra J. Cognitive and Neural Correlates of Loneliness and Wisdom during Emotional Bias. *Cereb Cortex*. 2021. |
