# Supplementary material for: Brief mindfulness coaching enhances selective attention in medical scientists: A pilot study
Source: PLoS One. 2025 Sep 12;20(9):e0330290. doi: 10.1371/journal.pone.0330290 (PMC12431218; doi:10.1371/journal.pone.0330290)
Supplement: Attachment — Submitted filename: additional_reviewer_comments.pdf [file pone.0330290.s003.pdf]

1. Page 5–Line 8: What is BrainE? Please define this term clearly upon first use. If it refers to a proprietary tool or software, consider presenting it as: "we have validated the reliability of BrainE, a [brief description, e.g., cognitive assessment] platform," rather than "the reliability of this BrainE platform."

2. 5–34: Using EEG measures is a large part of this study; could the authors clarify whether this constitutes a truly novel approach specifically in the context of interventions targeting health professionals? Even if it's not the first study to use EEG measures in this capacity, the inclusion of imaging techniques is great.

3. 5–42: The acronym "MSTP" is defined only at the end; it would improve clarity to define it upon first use.

4. 7–Figure 1:
   - What were the inclusion criteria for participants?
   - Consider whether this figure is the most effective use of space. Much of the information is redundant with the adjacent text or could be simplified. For example, listing the allocation group along with the number who did not receive the intervention may be unnecessary when the box is already labeled "Allocation to Waitlist Control Group (n = X)." It may be more useful to detail participant characteristics within each group (e.g., pre-clinical MSTP).
   - There appears to be a discrepancy: the figure indicates that 0 participants were excluded from analysis, but the text on line 6–23 states that some data were missing and excluded. Please clarify this inconsistency.

5. 7-19: When it says that the participants were divided into sub-groups, was that just for analysis purposes, or were they physically in separate groups? If so, what were the group numbers then? The faculty group would certainly be smaller. It's possible that the group size affected participation. Did both coaches coach at each session?

6. 8-15: The term "study arms" could be clarified…does it refer to the allocation groups or subgroups within them? Also, since the Garmin watch data were neither accessed by the research team nor used to provide feedback to participants, please elaborate on the intended role of the watches. Were they primarily intended as passive cues to support mindfulness engagement, similar to behavioral prompts (e.g., tying a string around a finger)?

7. 8-43: While the quote from Thich Nhat Hanh is compelling and thematically aligned, is it appropriate for inclusion in the body of an academic paper? Although his work is highly respected, it would strengthen the manuscript to support key points with peer-reviewed sources.

8. 9-39: Could the authors clarify why these particular quotes were chosen as illustrative examples? What criteria guided their selection?

9. Were there any subgroup differences? (e.g., was one effect stronger for junior faculty vs. graduate students)?

10. In the section describing the practices and activities, the use of numerous material-specific terms—often cited in quotations—can be distracting. While it's important to acknowledge the source material, many of these terms may be unnecessary for the reader's understanding. Instead, consider referencing key sources for those interested in the specific terminology or theoretical background. This would streamline the text while maintaining scholarly integrity.

11. The variety of mindfulness practices presented reads more like an eclectic collection than a purposefully sequenced curriculum. A clearer rationale for the selection and ordering of practices across the three sessions would enhance the intervention's coherence. Given the number of techniques used, a table outlining which practices targeted which outcomes (e.g., attention, emotional regulation) could help clarify the intervention's structure and theoretical underpinnings.

12. Finally, the tables are well-designed and clearly presented, a strength of the manuscript.
