# Supplementary material for: Brief mindfulness coaching enhances selective attention in medical scientists: A pilot study
Source: PLoS One. 2025 Sep 12;20(9):e0330290. doi: 10.1371/journal.pone.0330290 (PMC12431218; doi:10.1371/journal.pone.0330290)
Supplement: Attachment — Submitted filename: Responses to the Editor and Reviewers Comments.docx [file pone.0330290.s004.docx]

**Response to the Editor’s and the Reviewer’ Comments**

**Editor’s Comments:**

I have received feedback from an expert reviewer who raises significant concerns that must be addressed before the manuscript can proceed. Reviewer 1 notes that the sample size is underpowered, a judgement I share, and identifies a confounding factor complicating the interpretation of the results (see my detailed comments below). Given these substantial issues, I cannot accept the manuscript in its current form. We cannot proceed with a simple revision or a discussion of a few points, as there are fundamental problems. I recommend collecting additional data to increase the sample size; if this is not feasible, this limitation should be explicitly addressed. Therefore, I am rejecting the manuscript at this time. However, should you be able to address these concerns, I explicitly encourage resubmission. A revised manuscript should include a detailed point-by-point response letter addressing these issues.

**Response:** We thank the Editor for giving us an opportunity to resubmit our manuscript. After a substantial revision we have addressed all the Editor’s and the Reviewer’s comments in the manuscript that can be tracked in blue font in the revised submission.

Regarding additional data collection, we would like to share that the current study concluded last year in 2023 and is not funded anymore to further continue or to collect additional data. This limitation has now been explicitly mentioned in the Methods section.

**(-1-) Sample Size:**

The sample size (N = 43; 22 intervention group, 21 control group) is far too small, especially given the number of outcomes evaluated. As R1 notes, this raises significant concerns about the interpretation of non-significant findings, as it is unclear whether they reflect true null effects or insufficient statistical power. This limitation critically impacts the generalizability of the results and should have been addressed from the outset. While you acknowledge this issue in the discussion, merely stating it as a weakness is insufficient. I strongly recommend additional data collection to increase the sample size. If this is not feasible, a much more detailed analysis of the implications of the limited sample size is necessary to address this issue adequately.

**Response.** We thank the Editor for this comment and understand the concerns around generalizability. Due to limited resources, we are unable to collect any more data from the trainee physicians, as the current study concluded in the year 2023 and is not funded anymore. Following the Reviewer’s suggestion, we have further computed the Hedges’ g (bias corrected effect sizes) for each measure in the Results section (please see revised Tables 2 and 3) and have iterated at several instances (including the title) that this is a pilot study motivating future replication and on its own has limited generalizability.

**(-2-) Confounding Factors:**

For a valid comparison between groups, it is fundamental that they differ only in the critical experimental factor, with all other variables held constant. In this study, however, the intervention group not only received mindfulness coaching but also had access to Garmin wearable devices with lifestyle nudging. This introduces a significant confounding factor, rendering it impossible to discern whether the observed improvements in selective attention result from the mindfulness intervention or the effects of the wearable devices. Addressing this issue is essential to clarify the causal mechanisms underlying the findings.

**Response.** We thank the Editor for this comment, and have provided the following clarification in the Methods > Intervention section:

*“At the beginning of the study, all participants were also each provided a Garmin Vivosmart® 4 watch (a wearable device) to passively monitor lifestyle factors (physical activity, heart rate, sleep quality and stress) via a smartphone app coupled to the Garmin watch. The Garmin watches were provided to all participants across both study arms at the same time, so this digital intervention component did not differ between arms. All notifications were turned off on these devices with no feedback provided to individuals based on the watch data. The participants could engage with the app and look at their own data. This was neither encouraged nor tracked by the study team, and due to technical issues with the app the study team had no access to watch data regarding adherence or lifestyle fluctuations. Hence, only the timeline of the mindfulness coaching period differed between the two groups.”*

**(-3-) Theoretical underpinning**

**(--) restoration of attention**

The next issue concerns the rationale of the study, particularly the theoretical framework addressing how mindfulness is expected to lead to the restoration of attention, as evidenced by performance measures, self-reports, and psychophysiological data. Specifically, how do you conceptualize the working mechanism through which the intervention exerts its effects? For instance, does the mindfulness training induce an immediate cognitive state change, or do you expect that behavioral shifts emerge first, subsequently influencing cognitive processes? Also, which restorative-attention model is the theoretical underpinning that guides design and interpretation?

**Response.** We thank the Editor for this comment. In this revised version we have now integrated the theoretical framework both in the Introduction and the Discussion sections, as below:

Introduction -

…“*This decline is evident through slower reaction times, increased error rates, or greater work fluctuations, ultimately leading to reduced work efficiency (Barmack, 1939; Schumann et al., 2022). Based on prior reports on job demands and wellbeing, our theoretical assumption is that a high-pressure professional environment may affect the attentional capabilities and broader cognitive capacity of the physicians (Häusser & Mojzisch, 2017; Schumann et al., 2022).*

*The restoration of this reduction in attentional capabilities can be explained by the attentional-resource or energetic-capacity model (Schumann et al., 2022) that assumes depletion of a hypothetical reservoir of resources during work and recovery during rest. This model is based on Kaplan’s attention-restoration theory (ART) (Kaplan, 1995) that emphasize that “recovery is not only a function of leisure time but depends on the context where rest takes place.” ART is a psycho-sociological theory that explores the dynamics of recovery and stress within the real-world contexts of work and leisure. Hence, in the current study we propose an intervention program that would equip physicians with skills to use their period of rest actively to foster objectively measurable improvements in attentional or cognitive performance, as well as nurture subjective well-being.*

*… Subjective measures offer the advantage of providing insight into participants' personal assessments and feelings; however, they are susceptible to biases such as demand characteristics or tendencies toward certain self-presentation styles (Steghaus & Poth, 2024). To mitigate these biases, objective measures, including cognitive function measures and their associated neural mechanisms, can offer a more comprehensive understanding of the intervention program's effectiveness.*

… *Multidimensional cognitive assessments are also justified given that a recent metanalytic study reported that burnout may impair memory, attention and executive functioning and recommended mindfulness training as a protective remedy (Renaud & Lacroix, 2023). Our theoretical assumption is that the mindfulness training offered to physician trainees may facilitate improvements in present-moment-awareness and focus by cumulative practice over the intervention period that would be reflected in improved cognitive outcomes as well as concurrent neuroplasticity (Allen et al., 2012; Bauer et al., 2020). Furthermore, there is very limited literature exploring the impact of burnout on brain neuroplasticity among physicians (Abe et al., 2022; Bayes et al., 2021, Jaiswal et al., 2024). EEG measures can provide insights into neural plasticity induced by the intervention (Anguera et al., 2013; Mishra et al., 2014, 2015, 2020, 2021), which is a novel approach in the context of interventions for the healthcare community. In summary, we hypothesize that brief mindfulness training may equip physicians with mental skills that facilitate improvement of wellbeing, general cognition as well as neural changes that underlie those cognitive improvements.”*

Discussion -

*“The pattern of current findings fits with the energetic-resource model, which proposes that the state of resource depletion is reflected in the gradual decline of task performance speed over time (Schumann et al., 2022). Hence, we interpret the observed improvement in processing speed in the intervention group may be due to the replenishment of attentional resources by the practice of mindfulness. We also note that although we evaluated a battery of cognitive measures, selective attention results survived multiple comparisons correction across all tests and had large bias corrected between group effect size (Hedges’ g = 1.13). Nonetheless, our small study sample urges the need for follow-up studies with larger sample sizes.”*

**(--) expectable effects of three online mindfulness sessions?**

A related concern is the intervention itself: three online mindfulness training sessions spread over a 12-week period. Is it plausible, based on existing theory and evidence, to expect this dosage to produce a measurable effect? While I am not an expert in mindfulness training, it quite naturally raises the question of whether such limited exposure can reasonably be expected to result in detectable improvements. A stronger theoretical justification is needed to make this premise more convincing and to support the rationale behind the study design.

**Response:** The study began during the pandemic when strict health protection measures were still in place, particularly for healthcare professionals. Hence, the idea of administering mindfulness training via zoom originated. We resonate with the Editor/Reviewer’s concern that it is plausible based on existing theory and evidence that three online mindfulness training sessions spread over a 12-week period may be too low dose to produce any detectable improvements, which was also apparent in the subjective self-report outcomes. Yet, participants may imbibe important mindfulness skills in the three group coaching sessions that they may integrate in their daily life activities that may cumulate to replenishment of attention resources as observed in the post-intervention assessments. In this revised version we have provided a suitable theoretical justification for the mindfulness intervention, to make this premise more convincing and to support the rationale behind the study design. We have inserted the following at the end of the Methods > Intervention section:

“*We note that although the intervention structure in the current study is quite brief, it’s features resonate well with Kaplan’s ART (Kaplan, 1995) that integrates the dynamics of recovery and stress within the real-world contexts of work and leisure. The current intervention program encompassed teaching skills that have real-world applications. For example, coaches taught that all activities or daily actions offer opportunities to be in practice by bringing awareness to them, e.g. ‘washing dishes meditation’ and informal practice in our daily life by bringing awareness to whatever we may be doing in any given moment.*”

**(--) conceptual clarity**

Another issue concerns the conceptual clarity of both the intervention and the outcome measures. How do you define and operationalize the restored mind state, and what predictive framework informs the interpretation of your outcomes? Even within an exploratory approach, the study must be guided by hypotheses or at least plausible expectations about the pathways through which effects might emerge and why. To support a potential revision, I recommend consulting two theoretical works. The first is by Steghaus and Poth (doi:10.1371/journal.pone.0310034), which addresses the etiology and measurement of traits and states related to relaxation and tiredness. The second is a review by Schumann et al. (2022, doi:10.3389/fpsyg.2022.867978, particularly Table 2), which examines various theoretical concepts of mindfulness, restorative attention, and their measurement. These works could provide valuable insights into refining your conceptual framework and aligning your measures with the study’s theoretical objectives. To avoid conceptual ambiguity, it is essential to clearly define and distinguish the constructs used in this study, including which outcomes are chosen, why they are relevant, and what they indicate. Such alignment with theoretical foundations and hypotheses will strengthen both the validity and clarity of your findings.

**Response:** We thank the Editor for their questions; we have responded to these in the revised manuscript, and have also integrated the relevant references. The text revisions are quoted in the response to ‘theoretical underpinnings’ above.

**Reviewer 1 Comments**

Reviewer #1: This was a small randomized, controlled study sought to examine the effects of a mindfulness integrated psychological intervention on improvement of subjective well-being related behaviors, cognitive functioning and associated electrophysiological dynamics. The paper is interesting and well written with well stated scientific objectives and detailed data analysis section. This is a sophisticated and talented research team. There were only 43 subjects in total with group sizes of 22 vs. 21. The sample size within each group was powered to detect medium effect size pre vs. post difference (Cohen’s d >0.6) by t-test with beta power of 0.8 and alpha level of 0.05. This was a narrow focus for calculation considering the multiple endpoints and assessments as seen in Tables 2 and 3 and elsewhere throughout the manuscript. There appear to be no adjustment for multiple comparisons. Thus, perhaps one may suggest the reader should interpret the statistical significance statements with caution, perhaps in the limitations in the discussion. The investigators, to their credit, make reference to the small sample size at the top of page 22. Perhaps the study should be introduced as a pilot effort for a larger undertaking.

**Response:** We thank the Reviewer for their comments and raising the concern about multiple comparisons. Table 2 reports all the survey measures, wherein we did not observe any pre to post significant changes, hence we did not conduct any multiple comparisons correction for survey measures. However, we did conduct multiple comparison correction (False Discovery Rate) for all the cognitive measures in Table 3 and mention this further in the Discussion section. Additionally, we have inserted the Hedges’ g (bias corrected effect sizes) for each measure in Table 2 and 3 to enhance the objectivity of our results.

As the Reviewer suggests, we now further emphasize the need for future follow-up studies with larger sample sizes in the Discussion and have revised the title of the manuscript as *“Brief Mindfulness Coaching Enhances Selective Attention in Medical Scientists: A Pilot Study”*
