# Supplementary material for: Brief mindfulness coaching enhances selective attention in medical scientists: A pilot study
Source: PLoS One. 2025 Sep 12;20(9):e0330290. doi: 10.1371/journal.pone.0330290 (PMC12431218; doi:10.1371/journal.pone.0330290)
Supplement: Attachment — Submitted filename: Response to Reviewers.docx [file pone.0330290.s005.docx]

**Editorial Comments**

Please include the following items when submitting your revised manuscript:

- A rebuttal letter that responds to each point raised by the academic editor and reviewer(s). You should upload this letter as a separate file labeled 'Response to Reviewers'.
- A marked-up copy of your manuscript that highlights changes made to the original version. You should upload this as a separate file labeled 'Revised Manuscript with Track Changes'.
- An unmarked version of your revised paper without tracked changes. You should upload this as a separate file labeled 'Manuscript'.

*Response. As requested by the Editor, we have provided following items with this revised submission:*

*•	A rebuttal letter that responds to each point raised by the academic editor and reviewer(s) as a separate file labeled 'Response to Reviewers'.*

*•	A marked-up copy of your manuscript that highlights changes made to the original version as a separate file labeled 'Revised Manuscript with Track Changes'.*

*•	An unmarked version of your revised paper without tracked changes as a separate file labeled 'Manuscript'.*

Journal requirements:

When submitting your revision, we need you to address these additional requirements.

1. **Please ensure that your manuscript meets PLOS ONE's style requirements, including those for file naming. The PLOS ONE style templates can be found at**

https://journals.plos.org/plosone/s/file?id=wjVg/PLOSOne_formatting_sample_main_body.pdf and

https://journals.plos.org/plosone/s/file?id=ba62/PLOSOne_formatting_sample_title_authors_affiliations.pdf.

*Response. We have formatted the manuscript per PLOS ONE’s style requirements.*

**2. We note that the grant information you provided in the ‘Funding Information’ and ‘Financial Disclosure’ sections do not match.**

**When you resubmit, please ensure that you provide the correct grant numbers for the awards you received for your study in the ‘Funding Information’ section.**

*Response. We have now made these sections consistent regarding funding.*

3. Thank you for stating the following financial disclosure:

[This work was supported by a grant from the National Institutes of Health, NIH/NIGMS 1T32GM121318-01 (DMR, HHP), involved UCSD-MSTP trainees supported by NIH/NIGMS 5T32GM007198, and a Research Career Scientist Award from the Veterans Administration (BX005229 to HHP).].

Please state what role the funders took in the study. If the funders had no role, please state: ""The funders had no role in study design, data collection and analysis, decision to publish, or preparation of the manuscript.""

If this statement is not correct you must amend it as needed.

**Please include this amended Role of Funder statement in your cover letter; we will change the online submission form on your behalf.**

*Response. We have now stated that “The funders had no role in study design, data collection and analysis, decision to publish, or preparation of the manuscript.”*

4. Thank you for stating the following in the Acknowledgments Section of your manuscript:

[This work was supported by a grant from the National Institutes of Health, NIH/NIGMS 1T32GM121318-01 (DMR, HHP), involved UCSD-MSTP trainees supported by NIH/NIGMS 5T32GM007198, and a Research Career Scientist Award from the Veterans Administration (BX005229 to HHP). We thank Alankar Misra for software development of the BrainE platform and UCSD students who assisted with data collection. The BrainE software is copyrighted for commercial use (Regents of the University of California Copyright #SD2018-816) and free for research and educational purposes.]

We note that you have provided funding information that is not currently declared in your Funding Statement. However, funding information should not appear in the Acknowledgments section or other areas of your manuscript. We will only publish funding information present in the Funding Statement section of the online submission form.

**Please remove any funding-related text from the manuscript and let us know how you would like to update your Funding Statement. Currently, your Funding Statement reads as follows:**

*[This work was supported by a grant from the National Institutes of Health, NIH/NIGMS 1T32GM121318-01 (DMR, HHP), involved UCSD-MSTP trainees supported by NIH/NIGMS 5T32GM007198, and a Research Career Scientist Award from the Veterans Administration (BX005229 to HHP).]*

**Please include your amended statements within your cover letter; we will change the online submission form on your behalf.**

*Response. We have removed funding related text from the manuscript. The funding statement is correct.*

5. **Your ethics statement should only appear in the Methods section of your manuscript. If your ethics statement is written in any section besides the Methods, please move it to the Methods section and delete it from any other section. Please ensure that your ethics statement is included in your manuscript, as the ethics statement entered into the online submission form will not be published alongside your manuscript**.

*Response. Thank you for pointing this out. We have now ensured that the Ethics statement only appears in the Methods > Participants section.*

**6. In the online submission form, you indicated that your data will be submitted to a repository upon acceptance. We strongly recommend all authors deposit their data before acceptance, as the process can be lengthy and hold up publication timelines. Please note that, though access restrictions are acceptable now, your entire minimal dataset will need to be made freely accessible if your manuscript is accepted for publication. This policy applies to all data except where public deposition would breach compliance with the protocol approved by your research ethics board. If you are unable to adhere to our open data policy, please kindly revise your statement to explain your reasoning and we will seek the editor's input on an exemption**.

*Response. The dataset generated and analyzed in the current study is available from the datadryad.org repository:* [*https://datadryad.org/dataset/doi:10.5061/dryad.zpc866tfb*](https://datadryad.org/dataset/doi:10.5061/dryad.zpc866tfb)

*Please note that this link will be active once the study is published.*

**Review Comments to the Author**

Please use the space provided to explain your answers to the questions above. You may also include additional comments for the author, including concerns about dual publication, research ethics, or publication ethics. (Please upload your review as an attachment if it exceeds 20,000 characters)

**Reviewer #1: Dear Editor,**

Thanks for the opportunity to submit my review report titled “Brief Mindfulness Coaching Enhances Selective Attention in Medical Scientists: A Pilot Study “the hypothesis that brief mindfulness coaching (three 1.5-hour online group sessions over 12 weeks) can benefit medical scientists”

**Here are my comments:**

1. **The authors have included a section that captures detailed power analysis procedures conducted to arrive at a sample size of 43. Therefore, the study is powered to test the proposed hypothesis.**
2. **The intervention and control groups have been clearly defined, and detailed highlights of how the intervention group benefitted from the Brief Mindfulness Coaching have been provided.**

**Statistical Analysis: The use of the Chi-square test of independence for assessing associations between two categorical variables (gender and ethnicity), especially when most of the expected frequencies were less than 5 from Table 1, was not appropriate.**

**Ideally, Fisher's Exact test would have been better. Using a non-parametric Wilcoxon rank sum test for age comparisons between intervention and control groups was appropriate, but the authors should include why the non-parametric approach was adopted. Was it because there was a violation of normality and equal variance assumption required for a parametric two-sample t-test for age? How they tested for normality and equal variance is also missing under the Demographic characteristics section. They can equally provide a summary of how key statistical assumptions were tested to cater for all variables.**

**Please add a section called outcome measures (primary and secondary outcome measures) to provide proper context for the choice of statistical methods. You lumped all of them under data analysis. Kindly titled them outcome measures. The use of repeated measure ANOVA and Kruskal Wallis tests were appropriate.**

*Response. We thank the Reviewer for their thoughtful comments regarding the statistical approach in our study. Following the Reviewer’s suggestions we have updated the ‘Data Analysis’ section as ‘Outcome Measures and Data Analysis’. We have also allocated separate subsections for each outcome measures (Demographics, Self-report, Cognitive Assessment and Neural Assessment).*

*To address the Reviewer’s concerns regarding the demographic characteristics we would like to clarify that our study did not intend to find any association between categorical variables such as gender and ethnicity. Our decision to conduct Chi-square testing was simply to ensure that the two groups did not differ in either their distribution of gender or ethnicity. Since age was a non-categorical variable, we used the Wilcoxon rank sum test to compare age between groups.*

*We have further addressed the Reviewer’s concern by adding the following statements under the heading ‘Outcome Measures and Data Analysis’:*

*All the study measures were tested for normality using the Levene’s test. The normally distributed data were evaluated with parametric repeated measure analysis of variance (rm-ANOVA) for group x session interactions, while non-normal data were evaluated using the non-parametric equivalent of ANOVA, i.e., Kruskal-Wallis (KW) test (McKight & Najab, 2010). For any significant ANOVA results, post-hoc analyses were conducted within each group using either paired t-tests for normal data or the non-parametric equivalent Wilcoxon signed-rank test for non-normal data, and between-group effects in the ANOVA were further interrogated using either the two-sample t-test for normal data or otherwise the non-parametric Wilcoxon rank sum test was performed.*

**My major concern. This is an intervention study, but the results presented in Tables 2 and 3 do not show the intervention's true effect. Authors only presented pre-post for intervention and control groups, but the true effect size, which is a difference in difference, was not presented, making it difficult to determine the true effect of the intervention. That is the change in the intervention group minus the change in the control, including the corresponding confidence interval, would represent the true effect size, which is the difference in differences estimate.**

*Response. We thank the Reviewer for raising this concern. Following the Reviewer’s suggestion we have revised Tables 2 and 3 to show the confidence intervals and effect sizes based on the change in the intervention group minus the change in the control group.*

**Reviewer #2:**

Thank you for the opportunity to review this manuscript. The focus on supporting medical scientists, who are often under significant cognitive and emotional demands, through an abbreviated mindfulness intervention is both timely and important. The integration of EEG-based cognitive assessments alongside self-reported outcomes adds scientific rigor to the work, particularly in evaluating selective attention changes.

**That said, several aspects of the manuscript would benefit from further clarification and refinement to enhance its clarity, coherence, and impact.** **The rationale for the intervention design—including selection and ordering of mindfulness practices—could be made more explicit, and the use of extensive terms and quotations from external sources could be streamlined to improve readability. There is real potential here to contribute meaningfully to the literature on mindfulness interventions in high-stress training environments. With improved structural clarity and a more grounded theoretical justification for intervention components, this manuscript could be a valuable contribution to the field.**

*Response. We have now edited the manuscript for greater clarity, coherence and impact. The rationale for the intervention is now provided in the first paragraph of the intervention, and selection and ordering of practices has been explicitly stated. Extensive terms and quotes have also been streamlined.*

I appreciate the authors’ thoughtful engagement with this important topic and look forward to future developments in this line of work. Please see the attached comments.

**Page 5–Line 8: What is BrainE? Please define this term clearly upon first use. If it refers to a proprietary tool or software, consider presenting it as: “we have validated the reliability of BrainE, a [brief description, e.g., cognitive assessment] platform,” rather than “the reliability of this BrainE platform.”**

*Response.* *We thank the Reviewer for their comment. In this revision we have described BrainE with greater clarity as below:*

*“here we implemented a scalable platform, the Brain Engagement (BrainE©) tool, developed for free academic use by the Neural Engineering and Translation Labs (NEATLabs), for measuring core aspects of cognition. The BrainE© tool delivers standard cognitive assessments synchronized with neural recordings using electroencephalography (EEG) (Balasubramani et al., 2021; Misra et al., 2018). We have validated the reliability of BrainE© and shown its utility in measuring cognitive changes across the lifespan (Balasubramani, Diaz-Delgado, et al., 2022; Balasubramani, Walke, et al., 2022; G. Grennan et al., 2021, 2022; Mo et al., 2023; Ramanathan et al., 2024). We have also demonstrated its relevance in predicting mental health, well-being and burnout (G. K. Grennan et al., 2023; Kato et al., 2022; Nan et al., 2022, 2024; Shah et al., 2021).*

3. **5–34: Using EEG measures is a large part of this study; could the authors clarify whether this constitutes a truly novel approach specifically in the context of interventions targeting health professionals? Even if it’s not the first study to use EEG measures in this capacity, the inclusion of imaging techniques is great.**

*Response. We thank the Reviewer for this comment, and have modified the text for greater clarity as below:*

*“EEG measures can provide insights into neural plasticity induced by the intervention (Anguera et al., 2013; Mishra et al., 2014, 2015, 2020, 2021), which has not been applied in the context of interventions for the healthcare community apart from a couple of recent studies (Jaiswal et al., 2024, White et al., 2024).”*

4. **5–42: The acronym “MSTP” is defined only at the end; it would improve clarity to define it upon first use.**

*Response. We thank the Reviewer for this suggestion. The MSTP acronym has now been defined upon its first use as “participants were recruited from the University of California San Diego Medical Scientist Training Program (UCSD-MSTP)”.*

**4. 7–Figure 1:**

**- What were the inclusion criteria for participants?**

*Response. We have stated the inclusion criteria in the revised manuscript, “Inclusion criteria were current enrollment in the UCSD MSTP program or graduates of MD-PhD program.”*

**- Consider whether this figure is the most effective use of space. Much of the information is redundant with the adjacent text or could be simplified. For example, listing the allocation group along with the number who did not receive the intervention may be unnecessary when the box is already labeled “Allocation to Waitlist Control Group (n = X).” It may be more useful to detail participant characteristics within each group (e.g., pre-clinical MSTP).**

*Response. We totally agree with the Reviewer’s comment. However, it was a mandatory requirement requested by the Journal’s editorial team to provide a Consort Flowchart as Figure 1.*

**- There appears to be a discrepancy: the figure indicates that 0 participants were excluded from analysis, but the text on line 6–23 states that some data were missing and excluded. Please clarify this inconsistency.**

*Response. After the enrollment stage, no participants were excluded from allocation and analysis stages. For transparency, we have explicitly detailed how many participants had their data missing on what measures in the manuscript text. To avoid confusion, we have deleted the following sentence, “Missing data were excluded from analyses.” The final number of participants in both groups represents correct number of participants whose data were analyzed.*

**Were there any subgroup differences? (e.g., was one effect stronger for junior faculty vs. graduate students)?**

*Response. No there were no subgroup differences.*

**That said, several aspects of the manuscript would benefit from further clarification and refinement to enhance its clarity, coherence, and impact. The rationale for the intervention design—including selection and ordering of mindfulness practices—could be made more explicit, and the use of extensive terms and quotations from external sources could be streamlined to improve readability. There is real potential here to contribute meaningfully to the literature on mindfulness interventions in high-stress training environments. With improved structural clarity and a more grounded theoretical justification for intervention components, this manuscript could be a valuable contribution to the field.**

*Response. We have now edited the manuscript for greater clarity, coherence and impact. The rationale for the intervention is now provided in the first paragraph of the intervention, and selection and ordering of practices has been explicitly stated. Extensive terms and quotes have also been streamlined.*

**Additional Comments:**

•	**7-19: When it says that the participants were divided into sub-groups, was that just for analysis purposes, or were they physically in separate groups? If so, what were the group numbers then? The faculty group would certainly be smaller. It’s possible that the group size affected participation. Did both coaches coach at each session?**

*Response. We thank the reviewer for this comment and have now clarified these points in the manuscript as below:*

*“For the coaching sessions, participants were divided into sub-groups by career stage: pre-clinical MSTP students, MSTP graduate students, or clinician/junior faculty, so that there was an equivalent number of participants of 4-8 per sub-group allowing for adequate interaction time between participants, and also so that participants could easily connect with each other’s challenges given they were all in the same career-stage in a coaching session, making for more cohesive sessions where everyone felt similarly engaged.”*

*We have also clarified that the two coaches were always present in the coaching sessions.*

**•	8-15: The term “study arms” could be clarified…does it refer to the allocation groups or subgroups within them? Also, since the Garmin watch data were neither accessed by the research team nor used to provide feedback to participants, please elaborate on the intended role of the watches. Were they primarily intended as passive cues to support mindfulness engagement, similar to behavioral prompts (e.g., tying a string around a finger)?**

*Response. We have moved the Garmin watch description to the end of the Intervention section as both intervention and waitlist groups received the watches. We have removed the term study arms as it was confusing for the reviewer. Yes they were intended primarily as passive cues to support mindfulness engagement and we have clarified this in the text.*

**•	8-43: While the quote from Thich Nhat Hanh is compelling and thematically aligned, is it appropriate for inclusion in the body of an academic paper? Although his work is highly respected, it would strengthen the manuscript to support key points with peer-reviewed sources.**

*Response. We thank the reviewer for this comment and have removed the quote while keeping the appropriate reference.*

*•*	**9-39: Could the authors clarify why these particular quotes were chosen as illustrative examples? What criteria guided their selection?**

*Response. We have now clarified that these quotes were chosen to represent diverse individuals across different cultures and time periods that would resonate with the diverse participants, and all quotes were selected to share powerful common themes of personal empowerment, courage, inner transformation as a catalyst for outer change, and collective and ethical responsibility within our communities.*

*•*	**In the section describing the practices and activities, the use of numerous material-specific terms—often cited in quotations—can be distracting. While it’s important to acknowledge the source material, many of these terms may be unnecessary for the reader’s understanding. Instead, consider referencing key sources for those interested in the specific terminology or theoretical background. This would streamline the text while maintaining scholarly integrity.**

*Response. We thank the Reviewer for this comment and have streamlined the practices and activities section while acknowledging the appropriate citations.*

*•*	**The variety of mindfulness practices presented reads more like an eclectic collection than a purposefully sequenced curriculum. A clearer rationale for the selection and ordering of practices across the three sessions would enhance the intervention’s coherence. Given the** **number of techniques used, a table outlining which practices targeted which outcomes (e.g., attention, emotional regulation) could help clarify the intervention’s structure and theoretical underpinnings.**

*Response. We thank the reviewer for this comment, and have now provided a clearer rationale for the intervention and for the selection and ordering of practices. The overall rationale for the intervention is provided in the first paragraph of the intervention and its basis in Attention Restoration Theory. For the practices, we clarify that their selection and order was built from simpler, basic foundational practices to more complex forms that required engaging interaction, and all selected practices had much utility in real life - both work and leisure activities. We have now enumerated these practices better but have refrained from generating a table as many of the practices target overlapping outcomes.*
